# Characterize molecular signatures and establish a prognostic signature of gastric cancer by integrating single-cell RNA sequencing and bulk RNA sequencing

**DOI:** 10.1007/s12672-024-01168-w

**Published:** 2024-07-24

**Authors:** Zhiwei Wang, Zhiyan Weng, Luping Lin, Xianyi Wu, Wenju Liu, Yong Zhuang, Jinliang Jian, Changhua Zhuo

**Affiliations:** 1 Department of Gastrointestinal Surgical Oncology, Clinical Oncology School of Fujian Medical University, Fujian Cancer Hospital, Fuzhou, 350011 China; 2 Department of Endocrinology, the First Affiliated Hospital, Fujian Medical University, Fuzhou, 350005 China; 3 Department of Endocrinology, Binhai Campus of the First Affiliated Hospital, National Regional Medical Center, Fujian Medical University, Fuzhou, 350212 China; 4 Clinical Research Center for Metabolic Diseases of Fujian Province, the First Affiliated Hospital, Fujian Medical University, Fuzhou, 350005 China; 5 Department of Abdominal Medical Oncology, Clinical Oncology School of Fujian Medical University, Fujian Cancer Hospital, Fuzhou, 350011 China; 6 Fujian Key Laboratory of Translational Cancer Medicine, Fujian Provincial Key Laboratory of Tumor Biotherapy, Fuzhou, 350011 China

**Keywords:** Gastric cancer, Molecular signature, *COL4A1*, *FKBP10*, *RNASE1*, *SNCG*

## Abstract

Gastric cancer is a significant global health concern with complex molecular underpinnings influencing disease progression and patient outcomes. Various molecular drivers were reported, and these studies offered potential avenues for targeted therapies, biomarker discovery, and the development of precision medicine strategies. However, it was posed that the heterogeneity of the disease and the complexity of the molecular interactions are still challenging. By seamlessly integrating data from single-cell RNA sequencing (scRNA-seq) and bulk RNA sequencing (bulk RNA-seq), we embarked on characterizing molecular signatures and establishing a prognostic signature for this complex malignancy. We offered a holistic view of gene expression landscapes in gastric cancer, identified 226 candidate marker genes from 3 different dimensions, and unraveled key players’ risk stratification and treatment decision-making. The convergence of molecular insights in gastric cancer progression occurs at multiple biological scales simultaneously. The focal point of this study lies in developing a prognostic model, and we amalgamated four molecular signatures (*COL4A1*, *FKBP10*, *RNASE1*, *SNCG*) and three clinical parameters using advanced machine-learning techniques. The model showed high predictive accuracy, with the potential to revolutionize patient care by using clinical variables. This will strengthen the reliability of the model and enable personalized therapeutic strategies based on each patient’s unique molecular profile. In summary, our research sheds light on the molecular underpinnings of gastric cancer, culminating in a powerful prognostic tool for gastric cancer. With a firm foundation in biological insights and clinical implications, our study paves the way for future validations and underscores the potential of integrated molecular analysis in advancing precision oncology.

**Supplementary Information:**

The online version contains supplementary material available at 10.1007/s12672-024-01168-w.

## Introduction

Gastric cancer (GC) is a malignant tumor originating from gastric mucosal cells. Due to advances in early tumor screening and medical technologies, the mortality rate of gastric cancer has decreased compared to the past. However, it remains one of the primary causes of cancer-related deaths worldwide [1]. In recent years, more profound insights into the complex molecular genetic characteristics involved in gastric cancer occurrence and development have been provided with the continuous advancement of molecular genetics research, revealing the diversity and complexity of the pathogenesis of gastric cancer [2]. These studies not only elucidate the heterogeneity of gastric cancer leading to diverse clinical outcomes but also provide additional insights for subsequent personalized, targeted therapies.

Genetic alterations of GC are reflected in previous studies that provide insights into disease pathogenesis and candidate treatment targets. Recurrent genes such as extracellular calcium adhesive protein E-cadherin are crucial in cell-cell adhesion associated with the etiology of diffuse gastric cancer. Similarly, *TP53* mutation leads to the loss of conventional growth suppression and DNA repair capabilities [3]. High expression of HER2 in patients with copy number variations gives them a chance to be an immensely promising target for subsequent targeted therapeutic interventions [4]. Furthermore, other genetic mutations are linked to an elevated risk of gastric cancer, including *ATM* and *CHEK2* [5], involving an aberrant activation of multiple signaling pathways, including RAS, PI3K-Akt, and Wnt/β-catenin [6, 7]. In addition to intratumoral heterogeneity, external factors such as surrounding cells, extracellular matrix, and angiogenesis also profoundly influence tumor progression and response to treatments. Despite the recognized associations between clinic and molecular signatures in GC, previous genomic studies have largely focused on single targets.

According to the NCCN Guidelines (version 1, 2023), drug treatment plays an irreplaceable role for patients with intermediate to advanced gastric cancer and highlights targeted drug performance. Fluoropyrimidine and Cisplatin/Oxaplatin are recommended as primary systemic therapies for unresectable locally advanced, recurrent, or metastatic disease. Notably, it highlights that patients with HER2 expression can be targeted using trastuzumab, and Pembrolizumab/nivolumab can be administered based on the detection of MSI/MMR, PD-L1 immunohistochemical expression, or a high tumor mutational burden (TMB). It is evident that exploring the molecular genetic features and underlying mechanisms influencing outcomes in gastric cancer patients, along with understanding their immune microenvironment and inherent interactions, can further elucidate the characteristics of tumor cell initiation, progression, metastasis, and immune evasion. Patients can benefit from more accurate assessments, including identification of tumor signatures, evaluation of sensitivity to drugs, and personalized treatment.

To address these issues, we analyzed gastric cancer using scRNA-seq and bulk RNA-seq data to identify marker genes that have a significant impact on prognosis. Then, we developed a clinical riskScore model with superior predictive efficacy across both testing and validation datasets, which accurately predicts high-nomoScore patients with gastric cancer. Notably, we pinpointed four marker genes and three clinical features that exert a substantial influence on prognosis in gastric cancer and illustrated biological attributes and potential mechanisms underlying the high- and low-risk subgroups.

## Method

### Data acquisition and selection

We acquired gastric cancer scRNA sequencing data from the GEO database (http://www.ncbi.nlm.nih.gov/geo/), dataset ID: GSE167297, including samples from 5 patients, four normal tissue samples, and ten tumor tissue samples. Also, dataset ID: GSE62254 300 stomach adenocarcinoma (STAD) tumor samples, from which cancer tissue samples with prognosis information are extracted.

TCGA-STAD RNA-seq data and clinical information from the TCGA GDC database (https://portal.gdc.cancer.gov/projects/TCGA-STAD), along with TCGA-CDR [8] survival data. The dataset comprises 32 normal tissue samples and 375 tumor tissue samples. Tumor tissue samples lacking survival information and those with follow-up durations less than 30 days are excluded. Consequently, 339 tumor tissue samples are retained for further model development.

### Analysis of single-cell sequence data

Single-cell data analysis and annotation were conducted using Seurat (Version 4.3.0) [9] and singleR (Version 2.0.0) [10]. Data quality control criteria were defined as follows: 400 < nFeature_RNA < 5000, mitochondrial proportion (percent. mt) < 10%. Dimensionality reduction was performed using PCA, and batch correction was carried out using the harmony algorithm. The cell clustering resolution was set at 0.8. Marker genes for each cell subpopulation were identified using FindAllMarkers with parameter settings of min.pct = 0.25, and logfc.threshold = 0.7. Based on the marker genes of each cell subpopulation, the Single-Sample Gene Set Enrichment Analysis (ssGSEA) algorithm was applied to calculate ssGSEA enrichment scores for cell types in the entire set of STAD samples from TCGA. Wilcoxon rank-sum tests were employed to compare the differences in ssGSEA enrichment scores between tumor and normal samples for each cell type.

The R package clusterProfiler [11] (Version 4.7.1) was utilized to conduct functional enrichment analysis (GO and KEGG), CellChat package (Version 1.6.1) [12] for cell-cell communication analysis, and Pseudotime analysis was conducted using the monocle2 algorithm [13] (Version 2.26).

### Analysis of bulk RNA-seq data

#### Differential analysis

In the TCGA dataset, differential expression analysis was performed using the R package DESeq2 between tumor and normal samples(Version 1.36.0) [14], and FDR (corrected P-value) less than 0.05 and |log2FC|>1 were selected as the thresholds for filtering the differentially expressed genes.

#### WGCNA analysis

The TCGA dataset expression matrix (FPKM, Fragments Per Kilobase of transcript per Million mapped reads) data comprised expression values of 19,938 genes. We selected the top 10,000 genes based on the Median Absolute Deviation (MAD) for inclusion in the Weighted Gene Co-expression Network Analysis (WGCNA), which accounts for 50% of the total genes. The WGCNA package (Version 1.72-1) [15] was utilized for weighted gene co-expression network analysis (WGCNA) with sample grouping information (tumor, normal) serving as phenotype data. We determined an appropriate soft threshold value (power = 5) using pickSoft threshold. The relationship between these modules and phenotypes was investigated through correlation analysis, and gene modules with *P* < 0.05 were deemed significantly associated with gastric cancer.

#### Analysis of protein-protein interaction (PPI) protein interactions

A PPI network for candidate genes was established using the STRING database (https://cn.string-db.org/). Interactions with a combined score > 0.4 were chosen.

### Construction and evaluation of the prognostic signature

Tumor samples from the TCGA STAD dataset were randomly split into training and testing sets with a ratio of 6:4. Data from GEO were used as an external validation set. Subsequent modeling was performed on the training set, and the TCGA testing set, along with the GEO external validation set, was utilized to validate and assess the model.

We used univariate Cox regression analysis from the survival package (Version 3.4–0) [16] and conducted LASSO regression using the glmnet package [17] (version 2.0–18) in R. Survival-associated genes with significant correlation (*P* < 0.05) were then subjected to multivariate Cox regression analysis to establish the prognostic signature. The Riskscore calculation formula based on the prognostic signature is as follows: Riskscore = ∑βgene×Expgene. All samples were divided into high-risk (Riskscore ≥ median Riskscore) and low-risk (Riskscore < median Riskscore) groups based on the median Riskscore of the TCGA training cohort. The survival prognosis of the two groups was compared using Kaplan–Meier analysis. The receiver operating characteristic (ROC) curve for evaluating the predictive ability of the prognostic signature was plotted via R package survivalROC [18] (Version 1.0.3).

We used univariate and multivariate Cox analyses on both Riskscore and clinical characteristics to identify whether the prognostic signature was an independent risk factor using the survival package in R. The nomogram was constructed based on the significant characteristics in multivariate Cox analyses. Performing calibration curve analysis using the “rms” package [19] and drawing decision curve analysis (DCA) curves [18] using the “dcurves” package (Version 0.3.0) for further evaluating the clinical risk model.

### Correlation analysis for patients under different riskscore

Differential expression analysis was conducted between the High-risk and Low-risk subgroups using DESeq2. ClusterProfiler package [20] (Version 4.7.1.003) and GSVA algorithm [21] (Version 1.46.0) were used to perform GSEA analysis. Algorithms ESTIMATE, CIBERSORT (comprising 22 immune cell types), and ssGSEA (encompassing 28 immune cell types) were utilized to delve deeper into the disparities in the tumor microenvironment (TME) [22–24]. Waterfall plots were created using the R package maftools [25] (Version 2.14.0). OncoPredict package [26] (Version 0.2) was used for drug predictions, and CancerRxGene (GDSC) database, CTRP2 database (Cancer Therapeutics Response Portal), and TIDE database [27] were involved.

### Statistical analysis

The statistic analysis was conducted in R 4.2.1, and p value < 0.05 was considered statistically significant. We used t.test and Wilcoxon test for continuous variables and chi-square test for discrete variables.

## Results

### Single-cell profile in gastric cancer

**Fig. 1:**
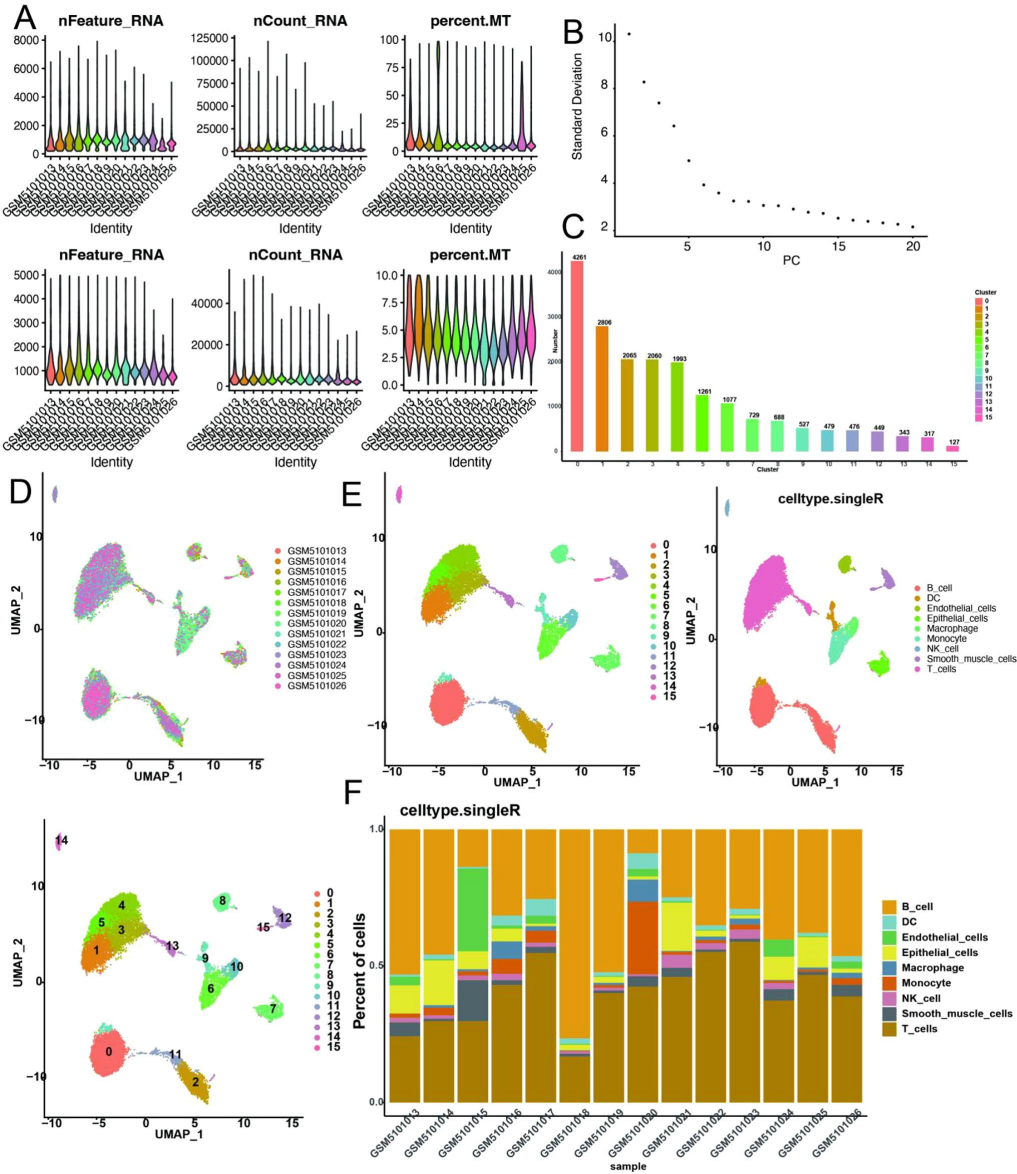
Single-cell transcriptome atlas of gastric cancer. **A** Cell Quality control and correction of samples (before: higher row; after: lower row). **B** Principal component analysis (PCA) and top 20 PCs are shown. **C** Number of cells in each cluster. **D**, **E** The UMAP(uniform manifold approximation and projection) plot: cells are classified into 16 clusters and annotated to 9 cell types in the end. **F** The percent of different types of cells in different samples

Analysis and correction of scRNA-seq data from gastric cancer (Fig. 1A) were performed and clinical information of there patients was summarized in supplemental file (Table.S1). 14 principal components were selected then for clustering and 16 cellclusters were obtained (Fig. 1B, D). Nine cell types were annotated in the end. Clusters 0 and 11 represented B cells. Clusters 1, 2, 3, 4, 5, and 13 were identified as T cells. Cluster 6 was identified as monocytes. Cluster 7 represented epithelial cells. Cluster 8 contained endothelial cells. Cluster 9 contained dendritic cells. Cluster 10 corresponded to macrophages. Cluster 14 consisted of NK cells. Smooth muscle cells were found in clusters 12 and 15 (Fig. 1E). Except for the T and B cell subgroups, the other cells displayed pronounced heterogeneity across various tumor samples (Fig. 1F).

**Fig. 2:**
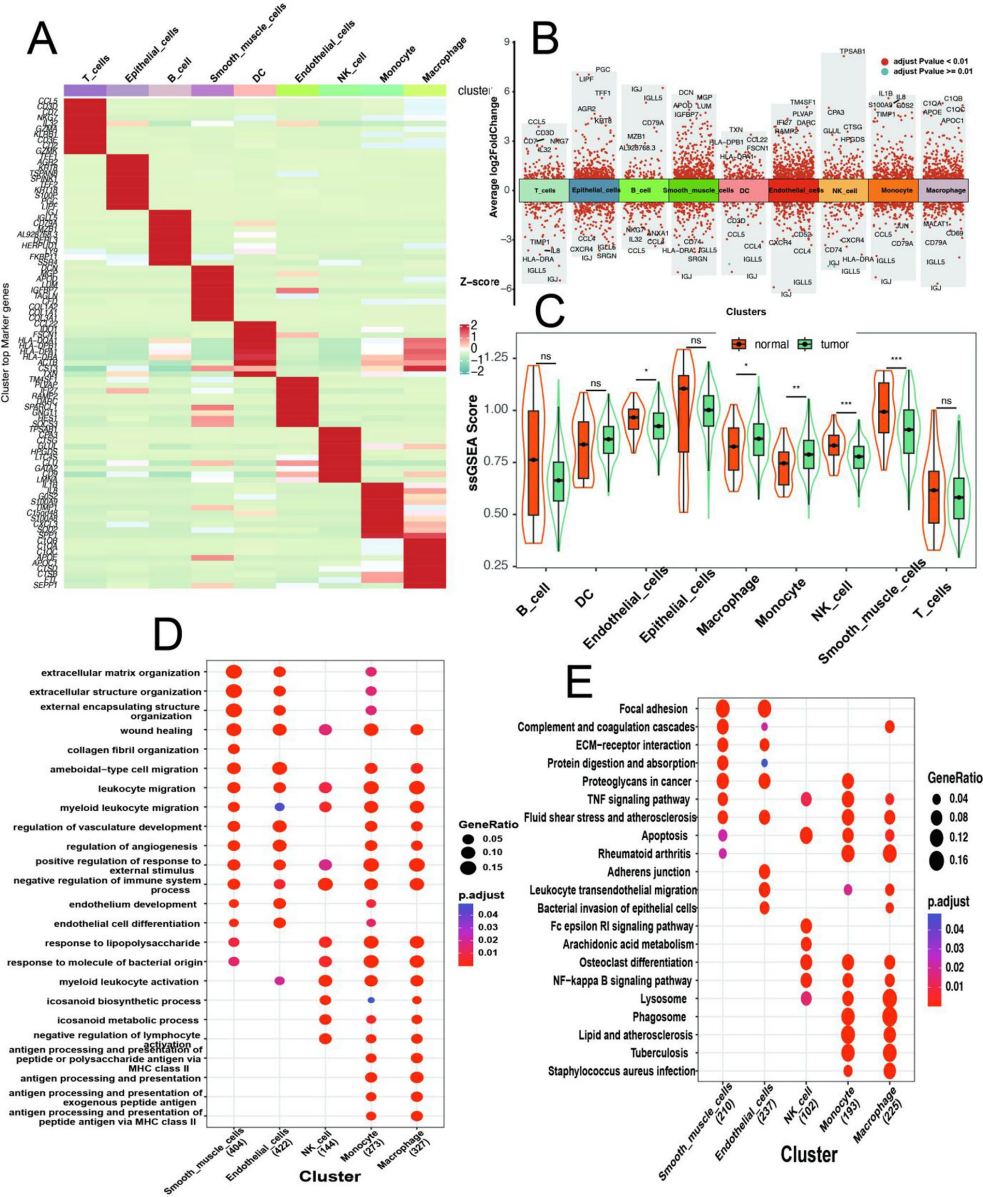
Clarification of key cell types and functional enrichment analysis of core marker genes. **A** Heatmap of top 10 marker genes in each type of cells. **B** Differentially expressed top genes in different cells. **C** Comparison of ssGSEA (single sample GSEA analysis) enrichment score between tumor and control samples. **D** The GO terms of BP categories enrichment of core marker genes. **E** KEGG enrichment of core marker genes

Through the utilization of the FindAllMarkers function, we identified a total of 1645 marker genes for nine cell subpopulations and marker genes for each cell type were shown in the plot (Fig. 2A, B). The ssGSEA algorithm was employed to compute ssGSEA scores for each cell type in samples from the TCGA STAD dataset. Furthermore, we compared ssGSEA scores for each cell type between tumor and normal samples. Notably, significant differences (*P* < 0.05) were detected in five distinct cell types: endothelial cells, monocytes, macrophages, NK cells, and smooth muscle cells. These five cell types were categorized as core cells (Fig. 2C). GO and KEGG functional enrichment analyses were performed on the marker genes associated with these five cell types (Fig. 2D, E). Notably, various functional pathways, such as the TNF signaling pathway, NF-kappa B signaling pathway, and ECM-receptor pathway, have been previously reported to be implicated in tumor progression and prognosis. We present corroborative evidence at the single-cell level in our research.

**Fig. 3:**
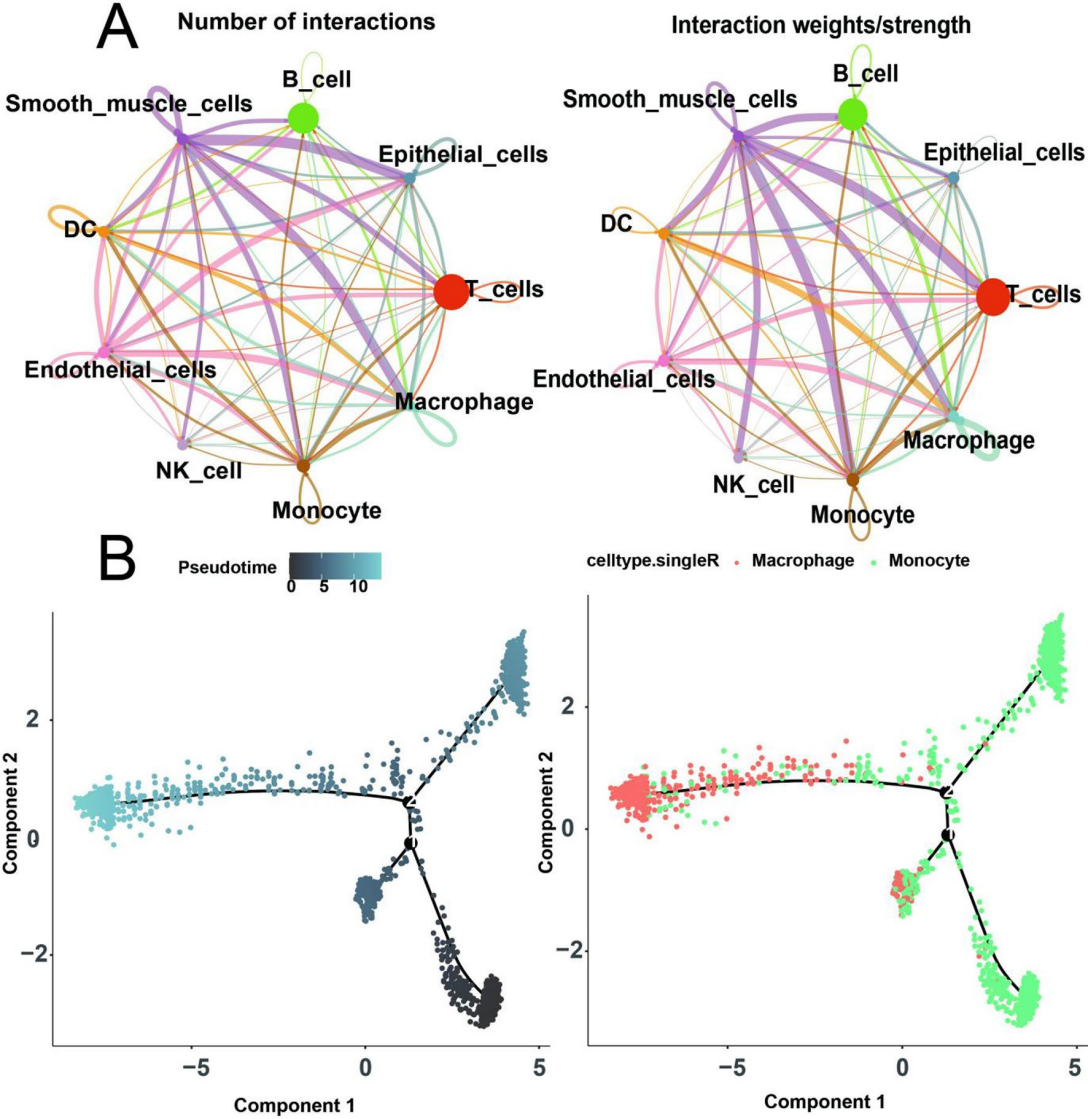
Cell-Cell communication and cell development trajectory between different celltypes. **A** Number (left) and weights (right) of interactions among different cells. **B** Trajectory analysis in monocytes and macrophages, including pseudotime ordering of cells (left) and cell types (right)

Cell-Cell communication analysis of the previously identified nine cell types was carried out utilizing CellChat [7] (Fig. 3A). The findings revealed elevated frequency and strength of interactions between smooth muscle cells and other cell types, and endothelial cells display the next highest level of interaction. Pseudo-time analysis of the monocyte and macrophage cell subtypes was conducted by using the monocle2 algorithm (Fig. 3B). It demonstrated the existence of two differentiation branches, with monocytes undergoing a unidirectional differentiation process towards macrophages.

### Identification and functional enrichment analysis of STAD- related genes in bulk RNAseq data

**Fig. 4:**
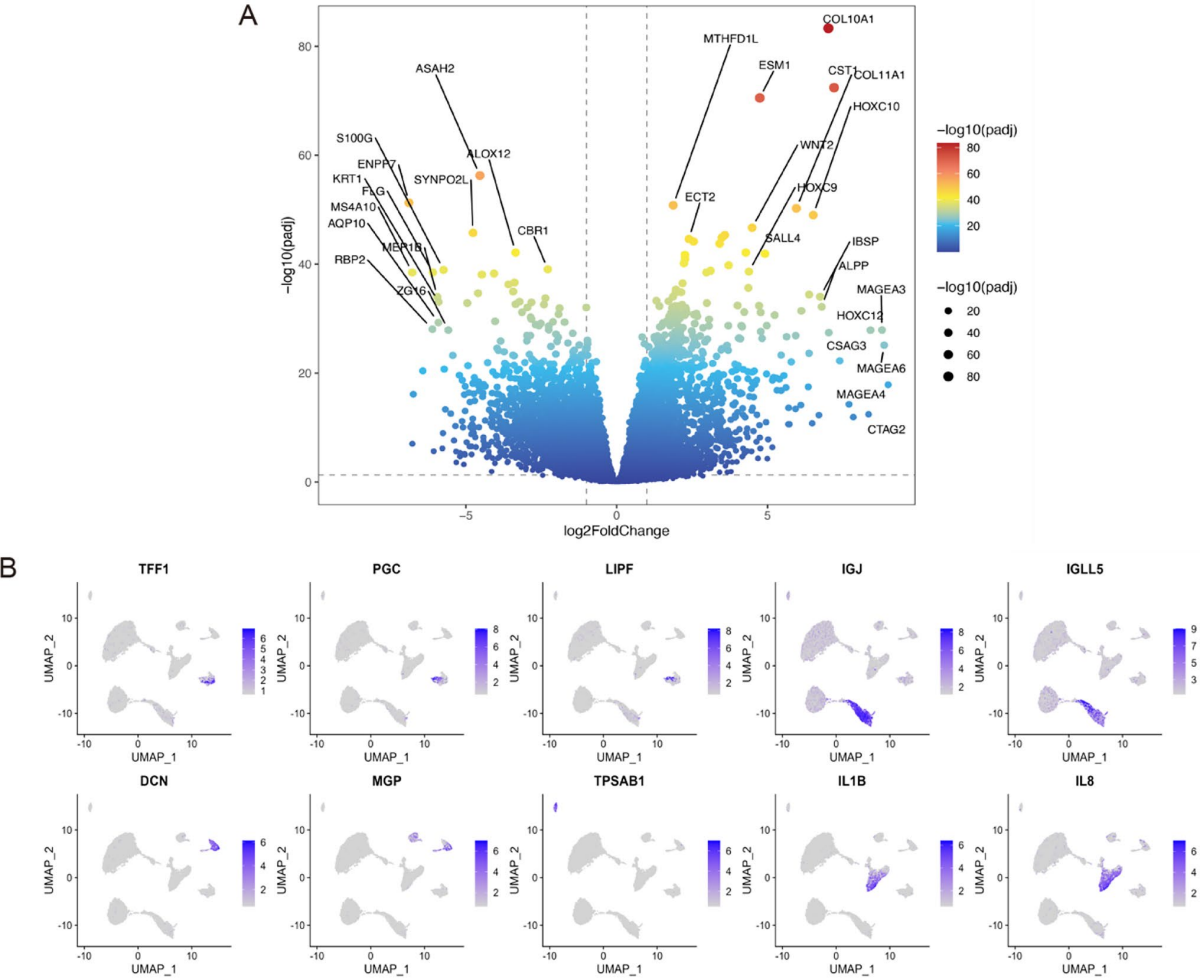
Differentially expressed candidate genes. **A** Differential analysis between tumor and control form TCGA-STAD bulk RNAseq samples, the top genes ranked by padj and logFC were labeled as top DEGs. The more red colors represent more significant difference. **B** The GC scRNAseq Umap (uniform manifold approximation and projection) plot of the marker genes associate with different cell types

Differential expression analysis was performed for all genes based on tumor and normal subgroup [FDR < 0.05; |log2FC|>0.58 (log2(1.5)], and 4516 differentially expressed genes were obtained, of which 2188 were up-regulated and 2328 down-regulated(Fig. 4A). The top 10 marker genes shown here, such as *TFF1* and *PGC*, are correlated to the gastric mucosal barrier and cellular protection, and the expression of *TFF1* was indicated to be reduced in some gastric cancer patients, particularly in some precancerous lesions, such as atypical hyperplasia(Fig. 4B). Other marker genes are also implicated in immune regulation and the tumor microenvironment.

**Fig. 5:**
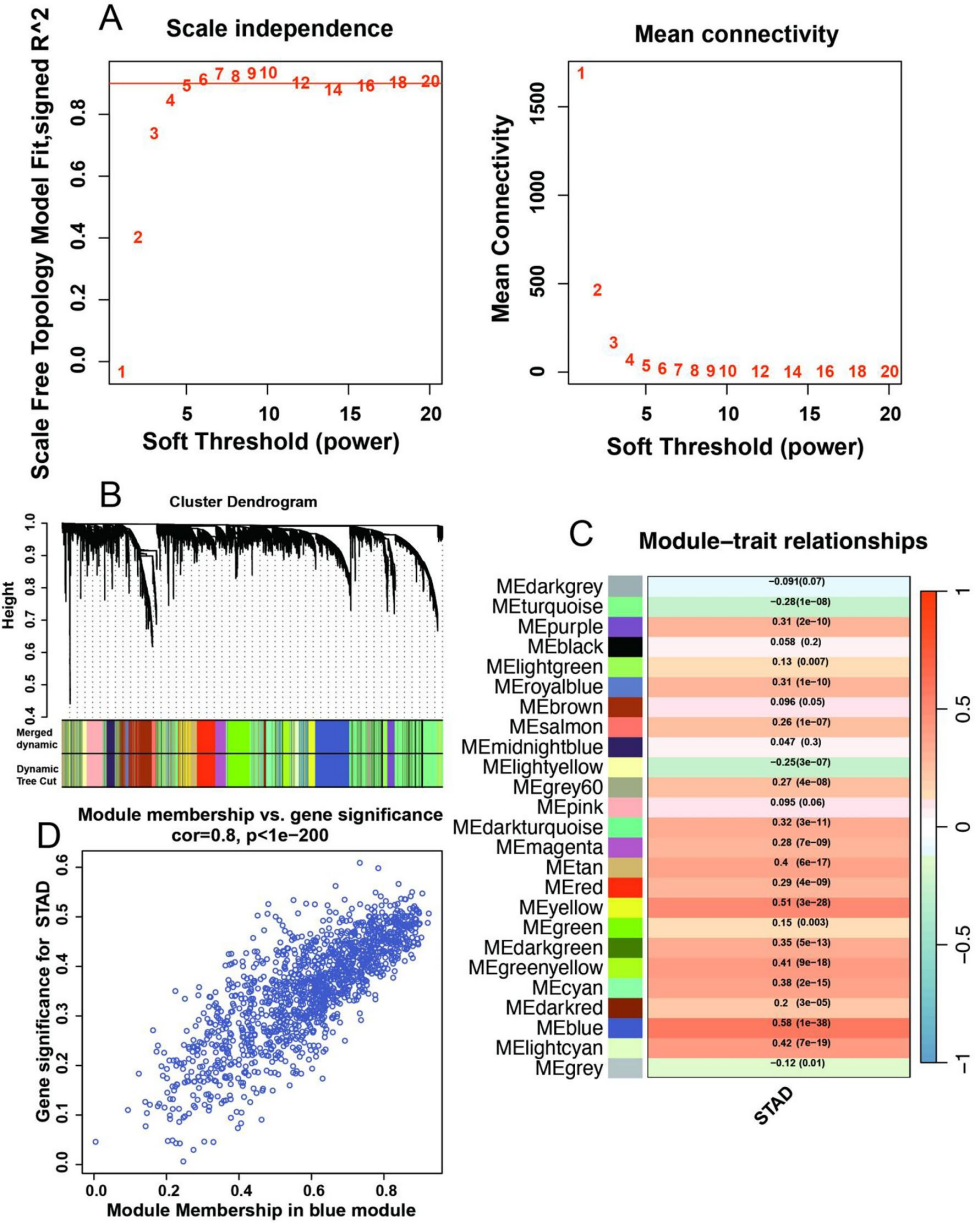
Screening STAD-related candidate genes through WGCNA. **A** Analysis of network topology for various soft-thresholding powers. **B** Clustering dendrogram of 25 co-expression gene modules with similar expression patterns. **C** Module-trait relationships of 25 gene modules. **D** The relationship between Module Membership (MM) in blue module and Gene Significance (GS) for STAD,.

Next, the sample groups were utilized as phenotype data for WGCNA analysis. Module detection was carried out via hierarchical clustering and dynamic tree-cutting functions (Fig. 5A). 25 modules were partitioned in total distinguished with different colors (Fig. 5B). 19 modules of all exhibited significant correlations with the STAD phenotype (*P* < 0.05), encompassing a total of 8186 genes (Fig. 5C). Particularly, the blue module showed the highest degree of correlation with the phenotype (correlation value = 0.8) (Fig. 5D).

**Fig. 6:**
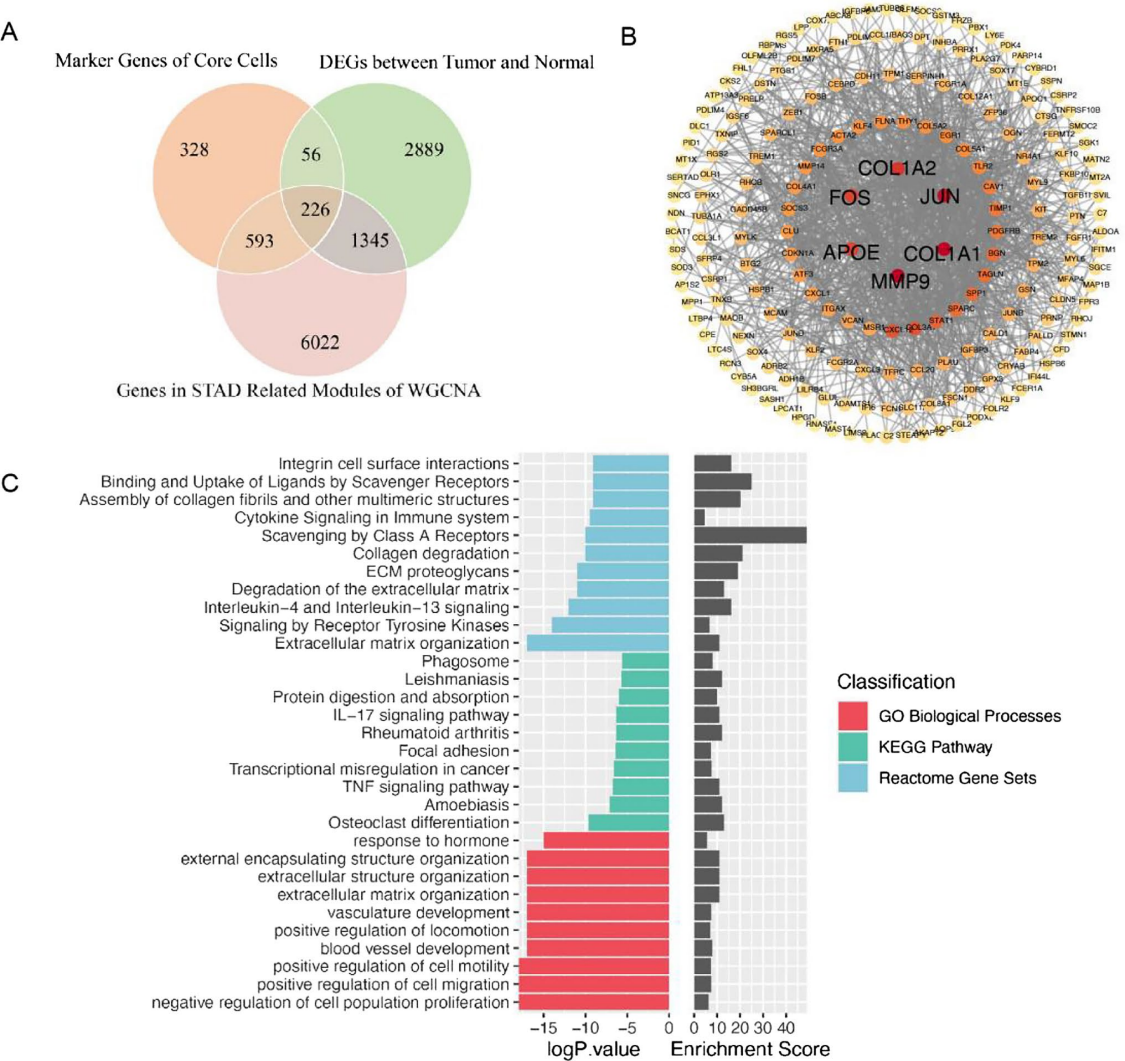
Discovery of candidate genes and functional analysis. **A** Venn plot of screening final candidate genes. **B** Protein-protein interaction network of final candidate genes. The color gradient, ranging from lighter to darker, represents the node’s degree from lower to higher. **C** Enrichment analysis for final candidates and top 10 enriched pathways are shown

We initially derived a set of 1203 marker genes specific to five distinct core cell types through the application of scRNA-seq analysis and identified 4516 genes displaying differential expression patterns using bulk RNA sequencing. Additionally, a comprehensive assessment of 8186 genes originating from 19 gene modules exhibiting significant associations with STAD was performed. The confluence of these three datasets resulted in the identification of a cohort of 226 potential candidate genes in the end (Fig. 6A). Furthermore, these candidate genes underwent scrutiny via protein-protein interaction (PPI) analysis (combined_score > 0.4) and resulted in a PPI network consisting of 198 nodes and 1188 edges (Fig. 6B). We note that key nodes such as *COL1A1*, *COL1A2*, *JUN*, *MMP9*, *APOE*, and *FOS* displayed strongest strength of interactions in the network, suggesting that these genes are key genes associated with other proteins in gastric cancer.

To further understand the biological functions of these differential genes in the gastric cancer, we did a comprehensive functional enrichment analysis of the aforementioned candidate genes by using the Metascape database. It revealed a significant enrichment of the 226 genes across 843 Gene Ontology (GO) bioprocess pathways, 72 KEGG pathways, and 107 Reactome pathways (Fig. 6C). Candidate genes are highly enriched in pathways including metabolism of substances, cellular interactions between intracellular and extracellular components and immune system. Scavenging by class A receptors showed the highest enrichment score.

### Identification of marker differentially expressed genes associated with prognosis and establishment of the prognostic signature

**Fig. 7:**
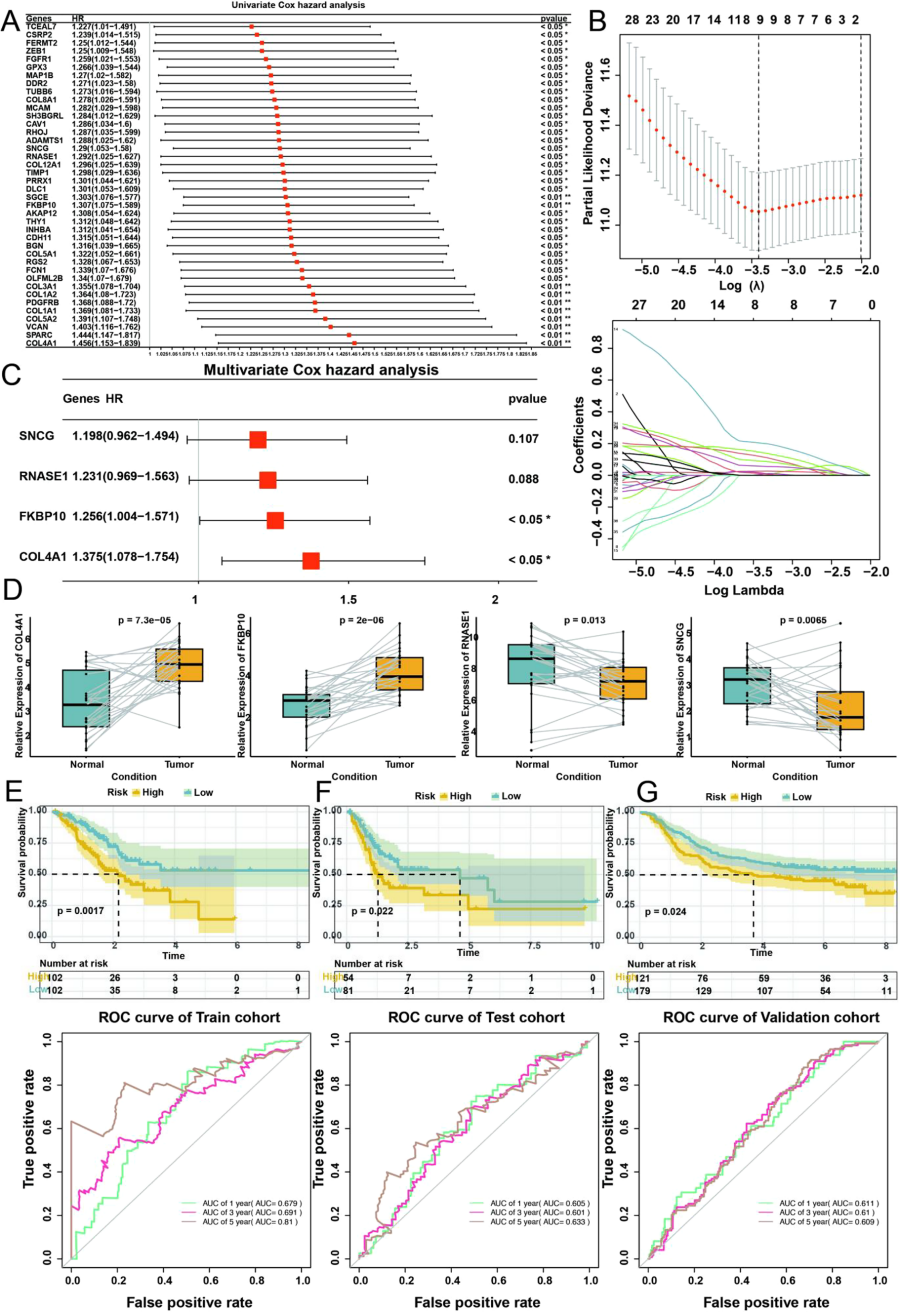
Construction and validation of gene-related risk model. **A** Univariate Cox regression analysis of final candidate genes. **B** Further filtering through LASSO regression analysis. **C** Multivariate Cox analysis. **D** Expression level of SNCG, RNASE1, FKBP10, CDL4A1 between tumor group and normal group in TCGA cohort. **E**–**G** Kaplan Meier plot and predictive survival rates of 1, 3, 5 years in training, test and validation cohort, respectively

In order to identify the characteristic DEGs that are associated with prognosis, we set up a training set to identify the candidate genes, a test set and an external validation set to evaluate and validate the model. In the TCGA training set, we executed a univariate Cox regression analysis by integrating the selected candidate genes with clinical features; 40 genes were identified that are significantly correlated to prognosis (Fig. 7A). We further conducted supplementary screening to discern nine genes that manifested a robust correlation with prognostic outcomes by using a LASSO 10-fold cross-validation approach ( Fig. 7B). The aforementioned nine genes were subjected to stepwise multivariable Cox regression analysis (Fig. 7C), resulting in the construction of a prognostic risk model comprised of four genes which highly expressed in gastric cancer and lead to poor prognosis (Fig. 7D). The mathematical formulation for computing the Riskscore is outlined as follows:

Riskscore = *COL4A1* * 0.318587101 + *FKBP10* * 0.227780778 + *RNASE1* * 0.207764548 + *SNCG* * 0.181031788.

TCGA samples were divided into high-risk and low-risk groups based on the median RiskScore. To avoid introducing biases into the results, we have provided the percentage of cases corresponding to each histological subtype in the baseline patient characteristics of the GSE62254 and TCGA-STAD cohort according to risk group in Supplementary Table S2. We used chi-square tests to compare the differences in Lauren’s histological subtype between samples from the high- and low-risk group and found no statistical difference. The application of Kaplan-Meier survival curves yielded evidence of a comparatively unfavorable prognosis for the high-risk group (p-value < 0.05) (Fig. 7E), and ROC curves [14] were subsequently employed to determine the area under the curve (AUC) values for 1, 3, and 5-year intervals and corresponding values of 0.679, 0.691, and 0.810, respectively. Upon subjecting the model to further validation and evaluation through the utilization of TCGA test data and an external validation dataset from the GEO database (Fig. 7F, G), consistent outcomes were obtained and AUC values for the 1, 3, and 5-year intervals within the TCGA test set were computed as 0.605, 0.601, and 0.633 respectively, while the corresponding values for the GEO external validation set were observed to be 0.611, 0.610, and 0.609.

**Fig. 8:**
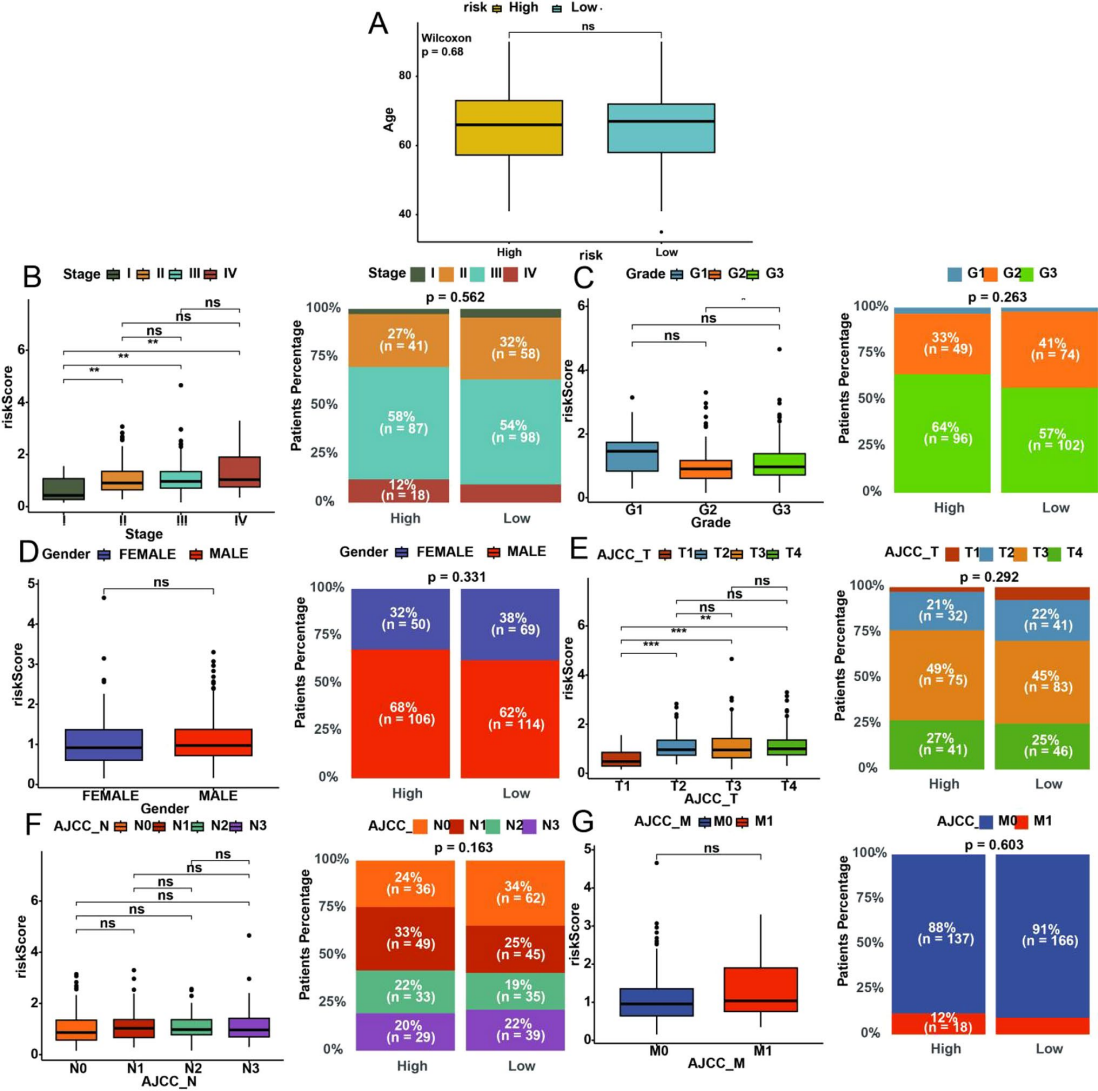
Clinical relevance of gene-related risk score. **A** Comparison of age between high- and low- risk subgroups. **B**–**G** Comparison of risk scores among different levels of clinical characteristics (subtitle of X axis) and proportion of clinical characteristics both in high- and low-risk subgroups

Then we explored the correlation between risk scores and pertinent clinical characteristics (Wilcox test), encompassing Age, Gender, Stage, Grade, and tumor TNM stage (Fig. 8). The risk score is associated with patient tumor stage, grade, size, and extent of the primary tumor. Patients in different risk subgroups do not exhibit significant clinical characteristic differences, further highlighting that the risk score is an independent prognostic factor influencing clinical outcomes in patients with gastric cancer.

### Construction of a nomogram based on the prognostic signature

**Fig. 9:**
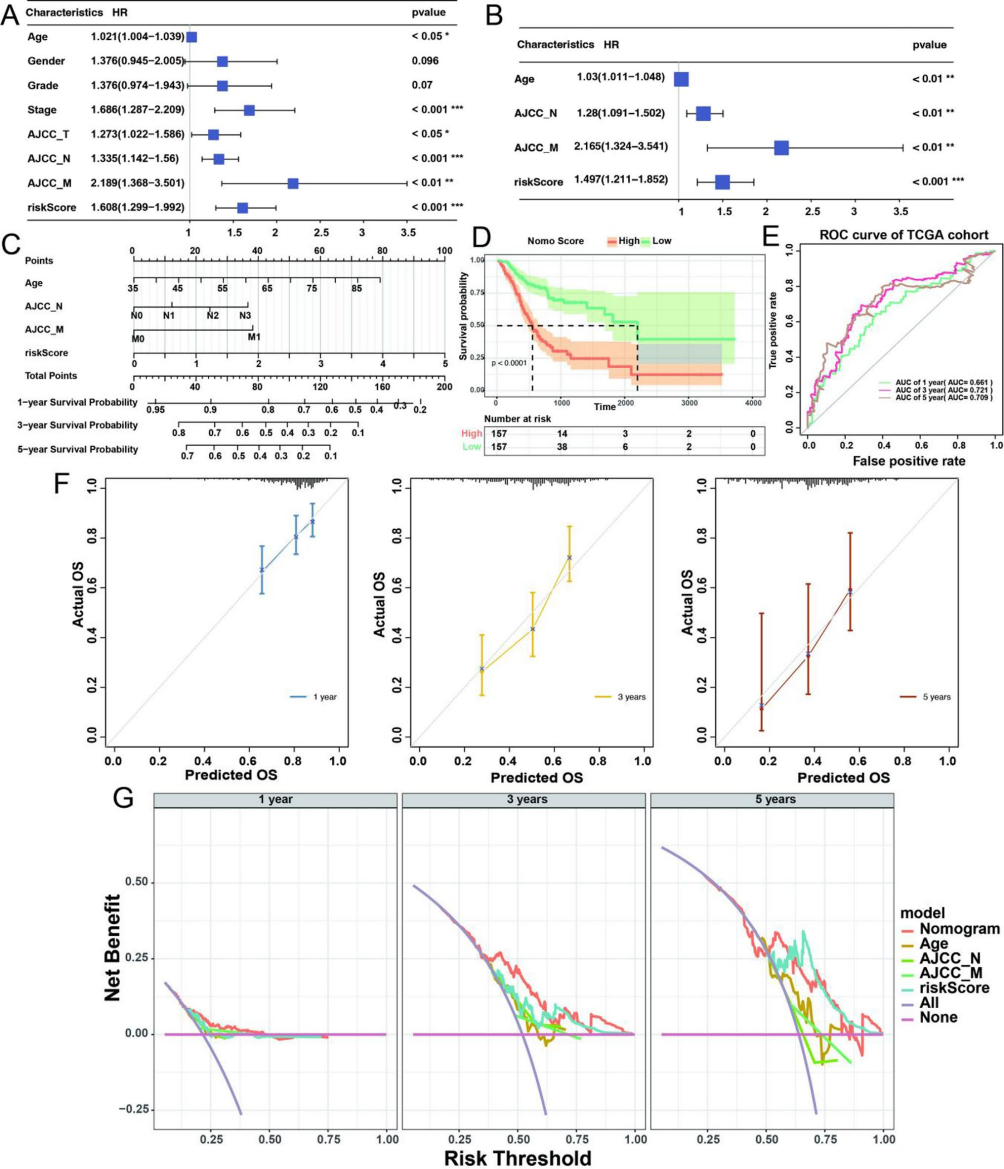
Construction and Evaluation of a Nomogram. **A** Univariate Cox regression analysis of riskScore and clinical characteristics. **B** Multivariate Cox regression analysis of riskScore and clinical characteristics. **C** Nomogram of clinical risk model. **D** Kaplan Meier plot of high- and low-nomoScore subgroups. **E** The ROC curve of 1, 3, 5 years of the Nomogram. **F** Calibration plots of the nomogram for predicting the probability of 1, 3, 5 years survival. **G** The DCA curves of the Nomogram compared for 1-, 3-, and 5-year overall survival in STAD, respectively

Through univariate Cox analysis of the RiskScore risk assessment and clinical features (Fig. 9A), it was observed that specific clinical characteristics such as Age, Stage, AJCC_T, AJCC_N, AJCC_M, and Riskscore were significantly associated with prognosis (p-value < 0.05). These prognostic factors were further subjected to stepwise multivariable Cox regression (Fig. 9B), resulting in the construction of a clinical risk model consisting of Age, AJCC_N, AJCC_M, and RiskScore, and the nomoScore for each patient, and the points of each clinical characteristic as depicted in the column chart (Fig. 9C, Supplementary Table S3), with a model C-index of 0.67. The nomoScore were calculated for TCGA STAD tumor samples based on the nomogram, which allowed the division of all samples into high- and low-nomoScore groups using the median nomoScore as a cutoff. The Kaplan-Meier curves were used to compare the survival differences between the two groups of patients (p-value < 0.001) (Fig. 9D, E), revealing poorer prognosis in the high-risk group. The ROC curves were employed to calculate the AUC values at 1, 3, and 5 years, resulting in values of 0.661, 0.721, and 0.709, respectively. Subsequently, the clinical risk model was evaluated using calibration and decision curve analysis (DCA) curves (Fig. 9F, G) [16], indicating favorable predictive performance of the model.

### The difference of molecular function between the high- and low-risk groups

**Fig. 10:**
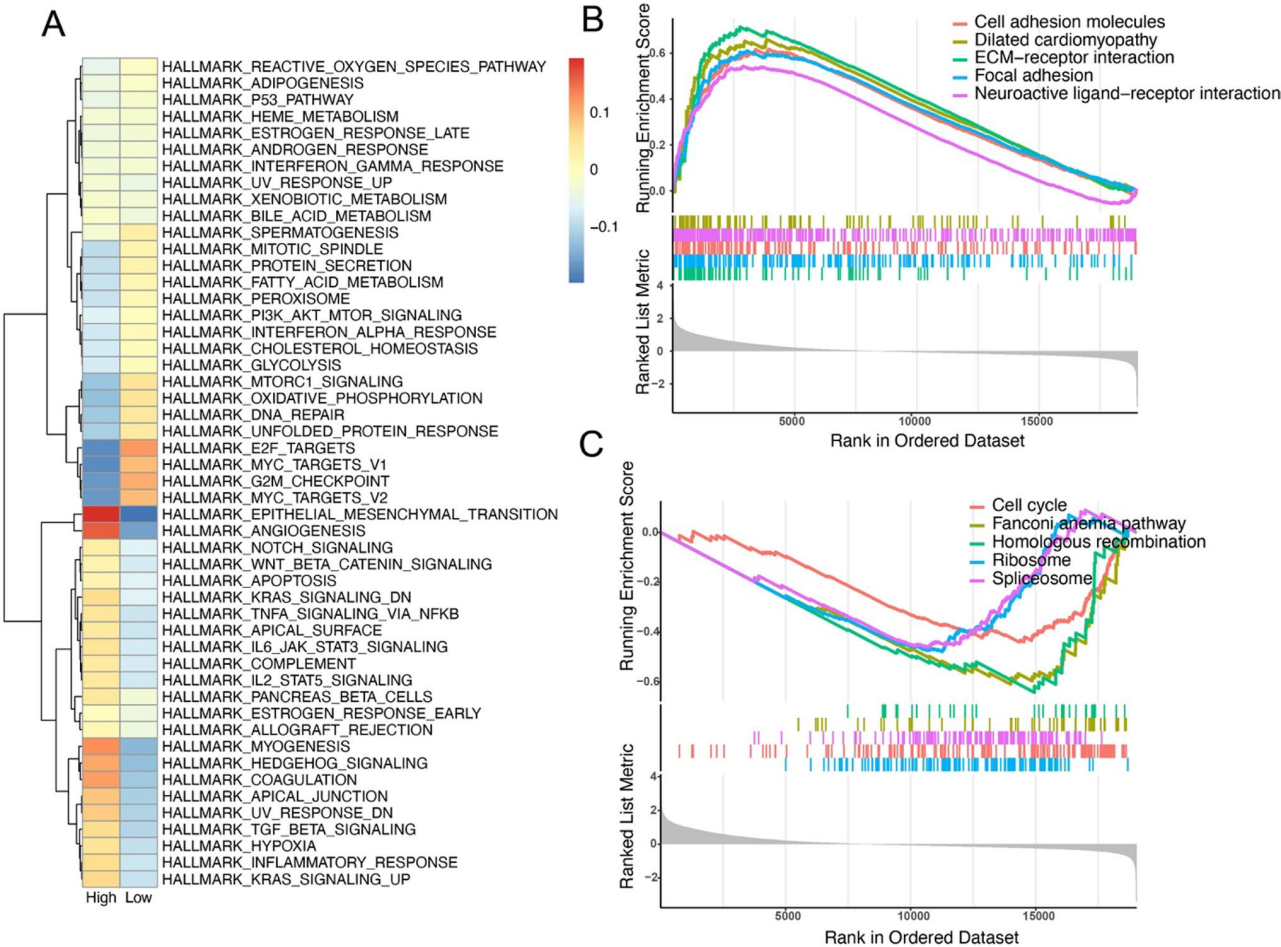
Enrichment analysis between high- and low-risk subgroups. **A** Enrichment analysis with hallmark gene sets. **B**, **C** Top 5 enriched pathways in high- and low-risk subgroups, respectively

Through the application of the GSVA algorithm, we conducted a comparison of enrichment scores for the HALLMARK pathway gene sets in the high- and low-risk subgroups (Fig. 10A), elucidating noteworthy variations in the enrichment scores of specific pathways between these subgroups and shedding light on the latent molecular mechanisms contributing to prognostic discrepancies in different risk subgroups. The most pronounced enrichment discrepancies between different groups are observed in the pathways of Epithelial-mesenchymal transition and angiogenesis, underscoring the divergent characteristics of different risk subgroups in terms of tumor metastasis and invasion. Concurrently, GSEA enrichment analysis of KEGG pathways was performed on samples from the high- and low-risk subgroups (Fig. 10B, C), elucidating that 56 KEGG pathways were enriched in the high-risk group and 19 KEGG pathways were enriched in the low-risk group. Intriguingly, the ECM-receptor interaction pathway was enriched in the high-risk group. It manifests the invasion and metastasis of gastric cancer involve the interaction between the extracellular matrix (ECM) and cell membrane receptors and has been demonstrated to play a significant role in the infiltration of gastric cancer.

### The Immune infiltration of M2-macrophages was different between high- and low-risk subgroups

**Fig. 11:**
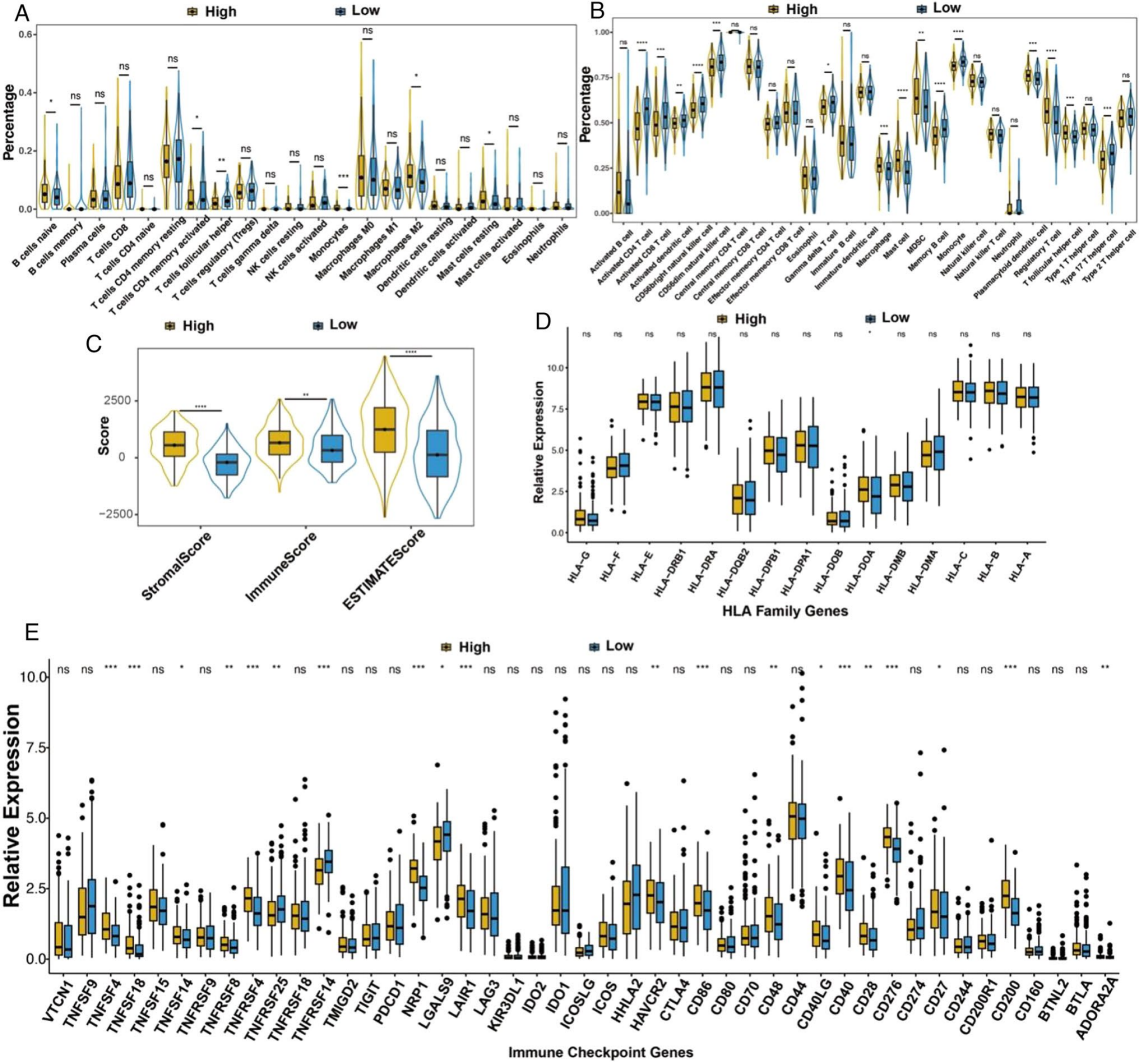
Correlation of RiskScorewith immune targets. **A**, **B** Abundance of different types of immune cells in subgroups with high and low RiskScore by using CIBERSORT (**A**) and ssGSEA (**B**). **C** Comparison of immune scores and stromal scores between high- and low-risk subgroups. **D** Expression of HLA family genes in different subgroups. **E** Expression of immune checkpoint genes in different subgroups

Immune infiltration analysis was performed on each sample using different algorithms, and the differences in immune cell abundance and immune matrix scores between high- and low-risk subgroups were compared using the Wilcox. test (Fig. 11A). The CIBERSORT analysis revealed disparities in 6 types of immune cells (p-value < 0.05). CIBERSORT analysis (Fig. 11A) revealed significant differences in six immune cell types (p-value < 0.05). Intriguingly, the expression of monocytes and macrophages M2 varied across different risk groups, suggesting a potential mechanism of immune suppression and tumor promotion in high- risk subgroups. Through ssGSEA analysis (Fig. 11B), significant differences (p-value < 0.05) in 15 immune cell types were observed between high and low-risk groups. ESTIMATE analysis (Fig. 11C) demonstrated that ESTIMATEScore, StromalScore, and ImmuneScore all exhibited significant differences (p-value < 0.05) between high and low-risk groups.

Extracting immune checkpoint gene and HLA family gene expression data from the subtype samples (Fig. 11D, E), we compared the differential gene expression between high and low- risk subgroups. Notably, we observed significant differences in expression levels of 20 immune checkpoint genes and 1 HLA family gene between the high and low- risk subgroups.

### Somatic mutation analysis

**Fig. 12:**
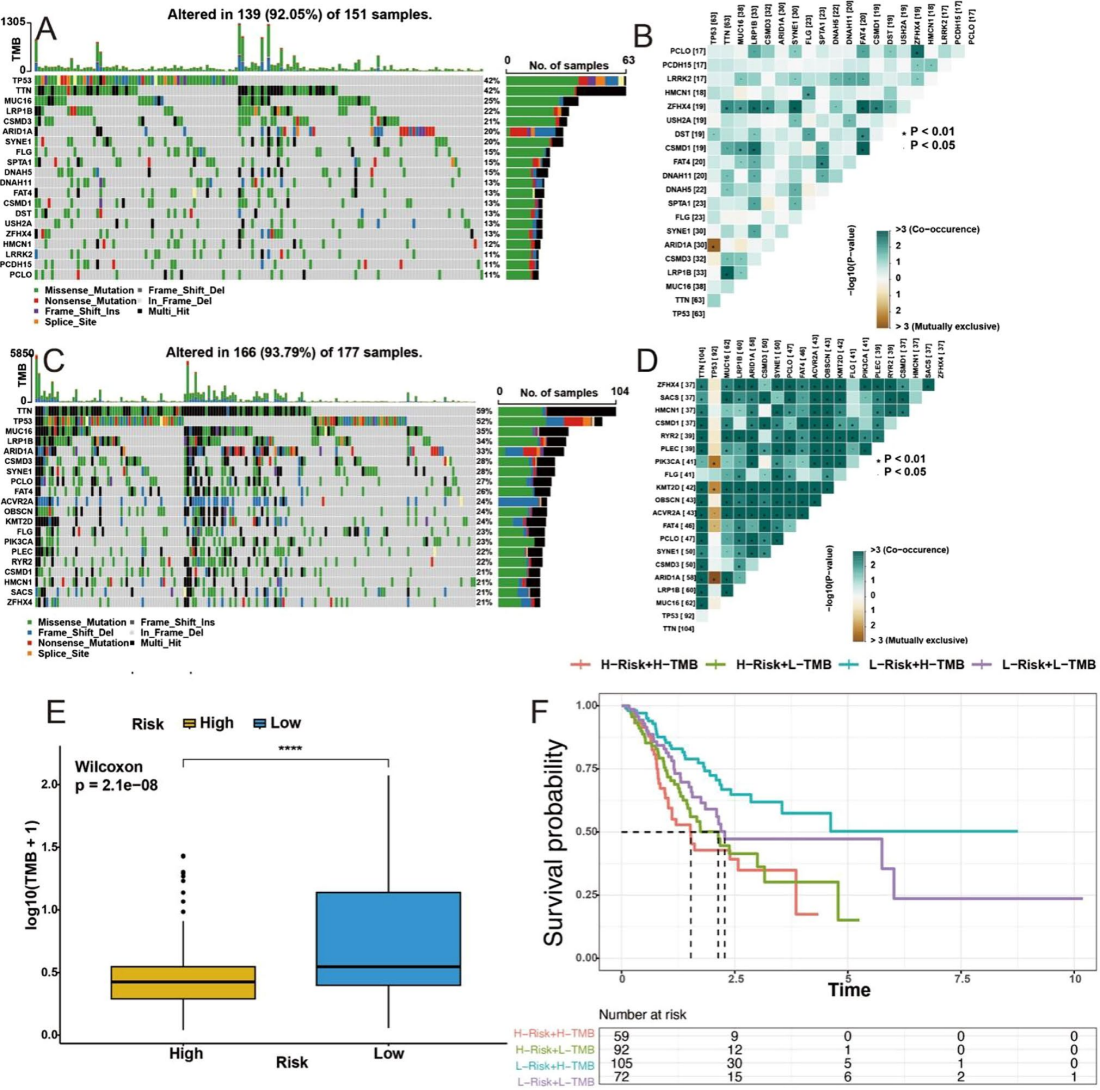
Genetic landscape in high- and low-risksubgroups. **A** Somatic mutations in high-risk subgroup. **B** Co-mutations in high-risk subgroup. **C** Somatic mutations in low-risk subgroup. **D** Co-mutations in low-risk subgroup. **E** Tumor mutational burden (TMB) in different subgroups. **F** Kaplan Meier plot of high- and low-risk subgroup together with different levels of TMB.

Based on somatic mutation data from the TCGA STAD database, we conducted a comparison of the top 20 genes with the highest mutation frequencies between high and low-risk subgroups, followed by an analysis of the co-mutation correlations among these top 20 genes (Fig. 12A–D). Through the Wilcoxon sum-rank test, a significant difference in Tumor Mutational Burden (TMB) was identified between high and low-risk subgroups (Fig. 12E). Compared to the high-risk group, the low-risk group exhibited higher TMB and a more intricate interplay among mutated genes. *TP53* mutations demonstrated more pronounced mutual exclusivity with other genes in both subgroups, with the most significant association with *ARID1A* mutations. Samples were categorized into high-TMB and low-TMB groups based on the median TMB, and clinical outcomes were analyzed through Kaplan-Meier curves between high and low-risksubgroups as well as between high- and low-TMB subgroups (Fig. 12F). The results indicated that TMB level was not significantly associated with patient prognosis under the conditions of RiskScore stratification.

### Drug sensitivity prediction and immunotherapy prediction

**Fig. 13:**
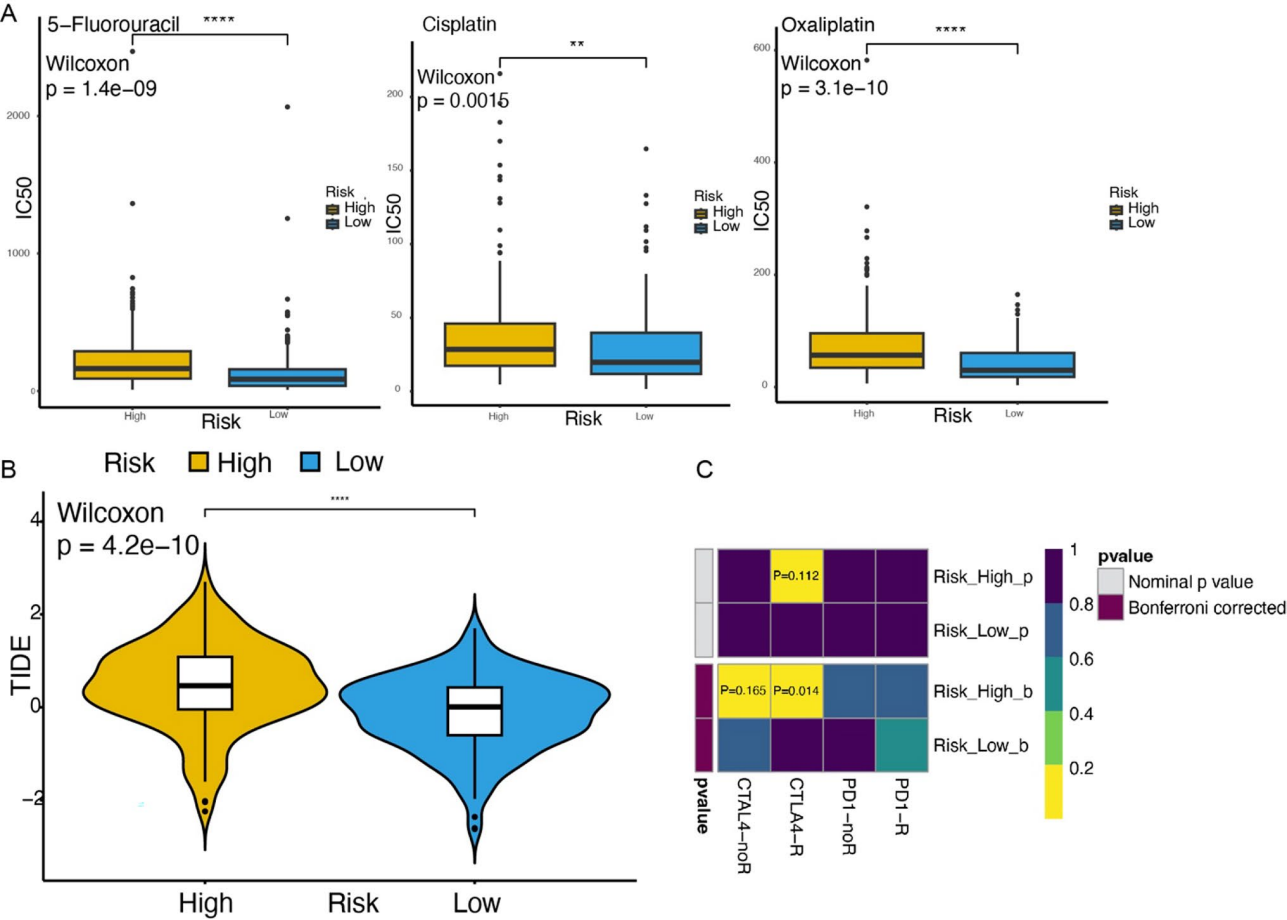
Therapeutic agents in high- and low-risk subgroups. **A** Drug Sensitivity in different subgroups, 5-Fluorouracil, Cisplatin and Oxaliplatin are shown in sequence. **B** Tumor immune dysfunction and exclusion (TIDE) score in different subgroups. **C** Clinical prediction of anti-PD1 and anti-CTLA4 in different subgroups

The sensitivity of each patient to chemotherapy drugs was estimated using the GDSC2 database and CTRP2 database (Fig. 13A). The Wilcoxon test was used to compare the differences in drug sensitivity between the high and low-risk subgroups. 139 of 198 drugs in the GDSC2 database exhibited significant differences in IC50 values between the high and low-risk subgroups. 320 of 545 drugs in the CTRP2 database showed significant differences in IC50 values between 2 subgroups. Sensitivity differences were observed for 5-Fluorouracil, Cisplatin, and Oxaliplatin, which are all part of the first-line systemic chemotherapy drugs for gastric cancer, indicating that the low-risk group is more sensitive to these first-line chemotherapy regimens.

To comprehensively analyze the therapeutic effects of immune-targeted drugs in different subgroups, we employed the TIDE database to predict the degree of benefit that patients may obtain from ICI treatment (Fig. 13B). We found a significant increase in TIDE scores among patients in the high-risk subgroup compared to the low-risk subgroup, indicating that patients in the high-risk subgroup might have a lower likelihood of benefiting from ICI treatment. Then, we predicted the response of patients to anti-PD1 and anti-CTLA4 treatments by using the Submap algorithm (Fig. 13C), revealing that patients in the high-nomoScore group might experience a more significant benefit from anti-CTLA4 treatment (Bonferroni-corrected *P* = 0.014).

## Discussion

We integrated gastric cancer single-cell data and bulk RNA-seq data and provided a comprehensive framework with a more stringent screening procedure of three distinct strategies during the process of feature gene selection. Initially, we conducted single-cell analysis and ssGSEA analysis to identify cells with differential expression, designating them as core cells and selecting marker genes from these core cells. Next, we proceeded to select differentially expressed genes from the bulk RNA-seq data and utilized WGCNA analysis to pinpoint module genes correlated with the gastric cancer phenotype additionally.

Continuing with the analysis, we identified 226 transcriptional candidate genes that are closely associated with the development, invasion, and immune microenvironment in GC. These candidate genes provide insights into the genetic signatures landscape in GC, further revealing their potential regulatory relationships and molecular mechanisms. Many of these genes have been reported in previous studies, and they serve as potential driver genes or regulatory elements that influence tumor progression through various pathways. Among the prominent six genes highlighted in the protein-protein interaction network, higher expression levels of *COL1A1* [28–31], *COL1A2* [32], and *APOE* [33, 34] have also been confirmed to contribute to shorter survival in gastric cancer.

To have a more transparent and more comprehensive understanding of the molecular and clinical features influencing prognosis in gastric cancer, we took clinical insights together with the characterization of genetic signatures and accurately identified four genes ( *COL4A1*, *FKBP10*, *RNASE1*, and *SNCG*) that are highly correlated with prognosis. Patients with high expression levels of these four genes tend to have poorer outcomes [35, 36]. *COL4A1* silence hampers the invasion, migration and epithelial-mesenchymal transition (EMT) of gastric cancer cells [35]. *FKBP10* is involved in protein folding, chaperone activity, and cellular processes related to collagen synthesis and extracellular matrix (ECM) remodeling [37, 38]. It has been indicated as a potential marker for lymph node metastasis in gastric cancer. *RNASE1* plays a significant role in the body’s innate immune response and contributes to the reduction of inflammation, ultimately leading to improved host defense and potential anti-cancer effects [39]. Previous studies have uncovered the potential of *RNASE1* as a mediator to reduce inflammation by breaking down surplus exRNAs [40, 41]. However, the significance of *RNASE1*’s involvement in gastric cancer has not been duly acknowledged. *SNCG* exhibits high expression levels in the gastric juice and serum of gastric cancer patients and contributes to the progression of the disease [42, 43]. Pathway enrichment analysis further confirmed that characteristics of the high-nomoScore subgroup are strongly associated with the invasion and metastasis of gastric cancer, involving interactions between the extracellular matrix (ECM) and cell membrane receptors.

In contrast to previous studies [44–46], our prognostic signature involves only 4 genes, aiming for a simpler and clearer approach. Having fewer genes in the prognostic model can enhance its straightforwardness and reduce the risk of overfitting, which can hinder the model’s performance on new data. What’s more, we employed univariate Cox and LASSO screening to identify prognosis-related genes. Then, by constructing a multivariate Cox model, we investigated the influence of these genes on gastric cancer prognosis. It is worth noting that in certain cases, there may exist intricate interactions between multiple genes, which cannot be fully captured by analyzing individual genes alone. In this regard, multivariate Cox models offer a superior solution, as they consider these complex interactions, thereby enhancing the explanatory and predictive capabilities of the model. Finnaly, the prognostic model in this study exhibited exceptional predictive performance as indicated by high AUC values in both the test set and the external validation set. Moreover, the results obtained from the external validation set showcased the model’s remarkable ability to generalize and apply to various sources data.

Furthermore, we demonstrated distinct immune microenvironment profiles in patients with different levels of RiskScore, which can influence the development and progression of tumors. It also provided more hints that patients under different levels of clinical risk scores may have different implications for the use of immunotherapies. Our results have indeed confirmed that tumors with higher RiskScore are more likely to benefit from anti-CTLA-4 (cytotoxic T-lymphocyte-associated protein 4) therapy, which is a type of immunotherapy that targets immune checkpoints to enhance the activity of T cells against cancer cells. Conversely, tumors in the low-risk group tend to be more sensitive to first-line chemotherapy drugs.

While the prognostic model has been constructed using standard procedures and its performance has been demonstrated with convincing evidence, it unfortunately still has some limitations. Firstly, in addition to TCGA, CGGA, and GEO, it is highly recommended that other databases, such as NoncoRNA, be included in our future studies. Secondly, although the methods have been utilized and validated in numerous research studies, it is imperative that we enhance our future studies with more advanced methodologies and technologies. Furthermore, the roles of the gastric cancer-related molecular signatures should be confirmed through in vitro and in vivo experiments. Our results may offer valuable insights for future research, focusing on the mechanistic process underlying GC.

In summary, our research enhanced our understanding of the complex interplay between genetic signatures, immune microenvironment, and clinical characteristics in GC. Meanwhile, it provided robust support for therapeutic decisions and contributed to the development of more personalized and effective therapeutic strategies for patients with GC.

### Electronic supplementary material

Supplementary Material 1.

Supplementary Material 2.

Supplementary Material 3.

## Data Availability

No datasets were generated or analysed during the current study.

## References

[1] Bray F, Ferlay J, Soerjomataram I, et al. Global cancer statistics 2018:GLOBOCAN estimates of incidence and mortality worldwide for 36 cancers in 185 countries. CA Cancer J Clin. 2018;68(6):394–424. 10.1007/s13304-023-01632-2. 30207593 10.1007/s13304-023-01632-2

[2] Smyth Elizabeth C, Nilsson M, Grabsch Heike I, et al. Gastric cancer. Lancet. 2020;396(10251):635–48. 10.1016/S0140-6736(20)31288-5. 32861308 10.1016/S0140-6736(20)31288-5

[3] Deng W, Hao Q, Vadgama J, et al. Wild-type TP53 predicts poor prognosis in patients with gastric cancer. J Cancer Sci Clin Ther. 2021;5(1):134–53. 10.26502/jcsct.50790107. 34950877 10.26502/jcsct.50790107 PMC8694034

[4] Gravalos C, Jimeno A. HER2 in gastric cancer: a new prognostic factor and a novel therapeutic target. Ann Oncol. 2008;19(9):1523–9. 10.1093/annonc/mdn169. 18441328 10.1093/annonc/mdn169

[5] Machlowska J, Kapusta P, Szlendak M, et al. Status of CHEK2 and p53 in patients with early-onset and conventional. Gastric cancer. Oncol Lett. 2021;21(5):348. 10.3892/ol.2021.12609. 33747205 10.3892/ol.2021.12609 PMC7967923

[6] Sihui T, Yada S, Shawna T, et al. AQP5 enriches for stem cells and cancer origins in the distal stomach. Nature. 2020;578(7795):437–43. 10.1038/s41586-020-1973-x. 32025032 10.1038/s41586-020-1973-x

[7] Mingli H, Shixuan Z, Shengwei X, et al. MicroRNAs and the PTEN/PI3K/Akt pathway in gastric cancer. Oncol Rep. 2019;41(3):1439–54. 10.3892/or.2019.6962. 30628706 10.3892/or.2019.6962

[8] Liu J, Lichtenberg T, Hoadley KA, et al. An integrated TCGA pan-cancer clinical data resource to drive high-quality survival outcome analytics. Cell. 2018;173(2):400-e41611. 10.1016/j.cell.2018.02.052. 29625055 10.1016/j.cell.2018.02.052 PMC6066282

[9] Hao Y, Hao S, Andersen-Nissen E, et al. Integrated analysis of multimodal single-cell data. Cell. 2021;184(13):3573–e358729. 10.1016/j.cell.2021.04.048. 34062119 10.1016/j.cell.2021.04.048 PMC8238499

[10] Aran D, Looney AP, Liu L, Wu E, Fong V, Hsu A, Chak S, Naikawadi RP, Wolters PJ, Abate AR, Butte AJ, Bhattacharya M. Reference-based analysis of lung single-cell sequencing reveals a transitional profibrotic macrophage. Nat Immunol. 2019;20:163–72. 10.1038/s41590-018-0276-y. 30643263 10.1038/s41590-018-0276-y PMC6340744

[11] Wu T, Hu E, Xu S, et al. clusterProfiler 4.0: a universal enrichment tool for interpreting omics data. Innovation. 2021. 10.1016/j.xinn.2021.100141. 34557778 10.1016/j.xinn.2021.100141 PMC8454663

[12] Jin S, Guerrero-Juarez CF, Zhang L, et al. Inference and analysis of cell-cell communication using CellChat. Nat Commun. 2021;12(1):1088. 10.1038/s41467-021-21246-9. 33597522 10.1038/s41467-021-21246-9 PMC7889871

[13] Qiu X, Mao Q, Tang Y, et al. Reversed graph embedding resolves complex single-cell trajectories. Nat Methods. 2017;14(10):979–82. 10.1038/nmeth.4402. 28825705 10.1038/nmeth.4402 PMC5764547

[14] Love MI, Huber W, Anders S. Moderated estimation of Fold change and dispersion for RNA-seq data with DESeq2. Genome Biol. 2014;15(12):550. 10.1186/s13059-014-0550-8. 25516281 10.1186/s13059-014-0550-8 PMC4302049

[15] Langfelder P, Horvath S. WGCNA: an R package for weighted correlation network analysis. BMC Bioinform. 2008;9:559. 10.1186/1471-2105-9-559. 10.1186/1471-2105-9-559 PMC2631488 19114008

[16] Therneau TM, Lumley T. Package ‘survival’[J]. R Top Doc. 2015;128(10):28–33.

[17] Friedman J, Hastie T, Tibshirani R. Regularization paths for generalized linear models via coordinate descent. J Stat Softw. 2010;33(1):1–22. 20808728 10.18637/jss.v033.i01 PMC2929880

[18] Heagerty PJ, Saha-Chaudhuri P, Saha-Chaudhuri MP. Package ‘survivalROC’[J]. San Francisco: GitHub; 2013.

[19] Harrell FE Jr, Harrell MFE Jr, Hmisc D. Package ‘rms’[J]. Vanderbilt Univ. 2017;229:Q8.

[20] Zhang J, Jin Z. (2023). _ggDCA: Calculate and Plot Decision Curve_. R package version 1.2.

[21] Hänzelmann S, Castelo R, Guinney J. GSVA: gene set variation analysis for microarray and RNA-seq data[J]. BMC Bioinformatics. 2013;14(1):1–15. 23323831 10.1186/1471-2105-14-7 PMC3618321

[22] Chen B, Khodadoust MS, Liu CL, et al. Profiling tumor infiltrating Immune cells with CIBERSORT[J]. Methods Mol Biol. 2018;1711:243. 29344893 10.1007/978-1-4939-7493-1_12 PMC5895181

[23] Kawada JI, Takeuchi S, Imai H, et al. Immune cell infiltration landscapes in pediatric acute myocarditis analyzed by CIBERSORT[J]. J Cardiol. 2020. 10.1016/j.jjcc.2020.08.004. 32891480 10.1016/j.jjcc.2020.08.004

[24] Yoshihara K, Shahmoradgoli M, Martínez E, et al. Inferring tumour purity and stromal and immune cell admixture from expression data[J]. Nat Commun. 2013;4(1):1–11. 10.1038/ncomms3612 PMC3826632 24113773

[25] Mayakonda A, Lin D, Assenov Y, Plass C, Koeffler PH. Maftools: efficient and comprehensive analysis of somatic variants in cancer. Genome Res. 2018. 10.1101/gr.239244.118. 30341162 10.1101/gr.239244.118 PMC6211645

[26] Maeser D, Gruener RF, Huang RS. oncoPredict: an R package for predicting in vivo or cancer patient drug response and biomarkers from cell line screening data. Brief Bioinform. 2021;22(6):bbab260. 10.1093/bib/bbab260. 34260682 10.1093/bib/bbab260 PMC8574972

[27] Roh W, Chen PL, Reuben A, et al. Integrated molecular analysis of tumor biopsies on sequential CTLA-4 and PD-1 blockade reveals markers of response and resistance. Sci Transl Med. 2017;9(379):3560. 10.1126/scitranslmed.aah356. 10.1126/scitranslmed.aah356 PMC5819607 28251903

[28] Yanlei Li, Ran S, Xiulan Z, et al. RUNX2 promotes malignant progression in gastric cancer by regulating COL1A1. Cancer Biomark. 2021;31(3):227–38. 10.3233/CBM-200472. 33896817 10.3233/CBM-200472 PMC12500011

[29] Cemre UM, Gulnihal O. Comprehensive bioinformatic analysis reveals a cancer-associated fibroblast gene signature as a poor prognostic factor and potential therapeutic target in gastric cancer. BMC Cancer. 2022;22(1):692. 10.1186/s12885-022-09736-5. 35739492 10.1186/s12885-022-09736-5 PMC9229147

[30] Yali W, Kun Z, XiuQiong C, et al. Bioinformatics analysis identifies COL1A1, THBS2 and SPP1 as potential predictors of patient prognosis and immunotherapy response in gastric cancer. Biosci Rep. 2021;41(1):BSR20202564. 10.1042/BSR20202564. 33345281 10.1042/BSR20202564 PMC7796188

[31] Shiping L, Long C, Jing Z, et al. A prognostic model based on the COL1A1-network in gastric cancer. Am J Transl Res. 2023;15(3):1640–53. eCollection 2023. 37056863 PMC10086874

[32] Li J, Ding Y, Li A. Identification of COL1A1 and COL1A2 as candidate prognostic factors in gastric cancer. World J Surg Oncol. 2016;14(1):297. 10.1186/s12957-016-1056-5. 27894325 10.1186/s12957-016-1056-5 PMC5126984

[33] Sakashita K, Tanaka F, Zhang X, et al. Clinical significance of ApoE expression in human gastric cancer. Oncol Rep. 2008;20(6):1313–9. 19020708

[34] Peiming Z, Qin L, Weiwei W, et al. Tumor-associated macrophages-derived exosomes promote the migration of gastric cancer cells by transfer of functional Apolipoprotein E. Cell Death Dis. 2018;9(4):434. 10.1038/s41419-018-0465-5. 29567987 10.1038/s41419-018-0465-5 PMC5864742

[35] Xijuan C, Tao S, Lina Q. Collagen type IV alpha 1 (COL4A1) silence hampers the invasion, migration and epithelial- mesenchymal transition (EMT) of gastric cancer cells through blocking hedgehog signaling pathway. Bioengineered. 2022;13(4):8972–81. 10.1080/21655979.2022.2053799. 35297303 10.1080/21655979.2022.2053799 PMC9161915

[36] Defeng L, Nannan W, Xin C, et al. Bioinformatics analysis suggests that COL4A1 may play an important role in gastric carcinoma recurrence. J Dig Dis. 2019;20(8):391–400. 10.1111/1751-2980.12758. 31069993 10.1111/1751-2980.12758

[37] Liang L, Kun Z, Jinhui Z, et al. Comprehensive evaluation of FKBP10 expression and its prognostic potential in gastric. cancer. Oncol Rep. 2019;42(2):615–28. 10.3892/or.2019.7195. 31233188 10.3892/or.2019.7195 PMC6609316

[38] LiBao G, Chuang Z, Ruoxi Y, et al. FKBP10 acts as a new biomarker for prognosis and lymph node metastasis of. Gastric cancer by bioinformatics analysis and in vitro experiments. Onco Targets Ther. 2020. 10.2147/OTT.S253154. 10.2147/OTT.S253154 PMC7395699 32801763

[39] Wang YN, Lee HH, Jiang Z, et al. Ribonuclease 1 enhances antitumor immunity against breast cancer by boosting T cell activation. Int J Biol Sci. 2023;19(10):2957–73. 10.7150/ijbs.84592. 37416781 10.7150/ijbs.84592 PMC10321278

[40] Zernecke A, Preissner KT. Extracellular ribonucleic acids (RNA) enter the stage in cardiovascular disease. Circ Res. 2016;118(3):469–79. 10.1161/CIRCRESAHA.115.307961. 26846641 10.1161/CIRCRESAHA.115.307961

[41] Fischer S, Cabrera-Fuentes HA, Noll T, Preissner KT. Impact of extracellular RNA on endothelial barrier function. Cell Tissue Res. 2014;355(3):635–45. 10.1007/s00441-014-1850-8. 24626811 10.1007/s00441-014-1850-8

[42] Pan Y, Zheng Y, Yang J, et al. A new biomarker for the early diagnosis of gastric cancer: gastric juice- and serum-derived SNCG. Future Oncol. 2022;18(28):3179–90. 10.2217/fon-2022-0253. 35947016 10.2217/fon-2022-0253

[43] Yi L, Qin P, Mingxia C, et al. Identification and validation of anoikis-associated gene SNCG as a prognostic biomarker in gastric cancer. Aging. 2023;15(7):2541–53. 10.18632/aging.204626. 36996495 10.18632/aging.204626 PMC10120907

[44] Cheong JH, Wang SC, Park S, et al. Development and validation of a prognostic and predictive 32-gene signature for gastric cancer. Nat Commun. 2022;13(1):774. 10.1038/s41467-022-28437-y. 35140202 10.1038/s41467-022-28437-y PMC8828873

[45] Huang S, Ma L, Lan B, Liu N, Nong W, Huang Z. Comprehensive analysis of prognostic genes in gastric cancer. Aging. 2021;13(20):23637–51. 10.18632/aging.203638. 34686626 10.18632/aging.203638 PMC8580339

[46] Chang J, Wu H, Wu J, et al. Constructing a novel mitochondrial-related gene signature for evaluating the tumor immune microenvironment and predicting survival in stomach adenocarcinoma. J Transl Med. 2023;21(1):191. 10.1186/s12967-023-04033-6. 36915111 10.1186/s12967-023-04033-6 PMC10012538

